# Genomic adaptation of *Burkholderia anthina* to glyphosate uncovers a novel herbicide resistance mechanism

**DOI:** 10.1111/1758-2229.13184

**Published:** 2023-06-13

**Authors:** Inge Schwedt, Madeline Collignon, Carolin Mittelstädt, Florian Giudici, Johanna Rapp, Janek Meißner, Hannes Link, Robert Hertel, Fabian M. Commichau

**Affiliations:** ^1^ FG Synthetic Microbiology, Institute for Biotechnology BTU Cottbus‐Senftenberg Senftenberg Germany; ^2^ FG Molecular Microbiology, Institute of Biology University of Hohenheim Stuttgart Germany; ^3^ Interfaculty Institute for Microbiology and Infection Medicine Tübingen University of Tübingen, Bacterial Metabolomics Tübingen Germany; ^4^ Department of General Microbiology, Institute for Microbiology and Genetics University of Goettingen Göttingen Germany; ^5^ Department of Genomic and Applied Microbiology, Institute for Microbiology and Genetics University of Goettingen Göttingen Germany

## Abstract

Glyphosate (GS) specifically inhibits the 5‐enolpyruvyl‐shikimate‐3‐phosphate (EPSP) synthase that converts phosphoenolpyruvate (PEP) and shikimate‐3‐phosphate to EPSP in the shikimate pathway of bacteria and other organisms. The inhibition of the EPSP synthase depletes the cell of the EPSP‐derived aromatic amino acids as well as of folate and quinones. A variety of mechanisms (e.g., EPSP synthase modification) has been described that confer GS resistance to bacteria. Here, we show that the *Burkholderia anthina* strain DSM 16086 quickly evolves GS resistance by the acquisition of mutations in the *ppsR* gene. *ppsR* codes for the pyruvate/ortho‐P_i_ dikinase PpsR that physically interacts and regulates the activity of the PEP synthetase PpsA. The mutational inactivation of *ppsR* causes an increase in the cellular PEP concentration, thereby abolishing the inhibition of the EPSP synthase by GS that competes with PEP for binding to the enzyme. Since the overexpression of the *Escherichia coli ppsA* gene in *Bacillus subtilis* and *E. coli* did not increase GS resistance in these organisms, the mutational inactivation of the *ppsR* gene resulting in PpsA overactivity is a GS resistance mechanism that is probably unique to *B. anthina*.

## INTRODUCTION

**FIGURE 1:**
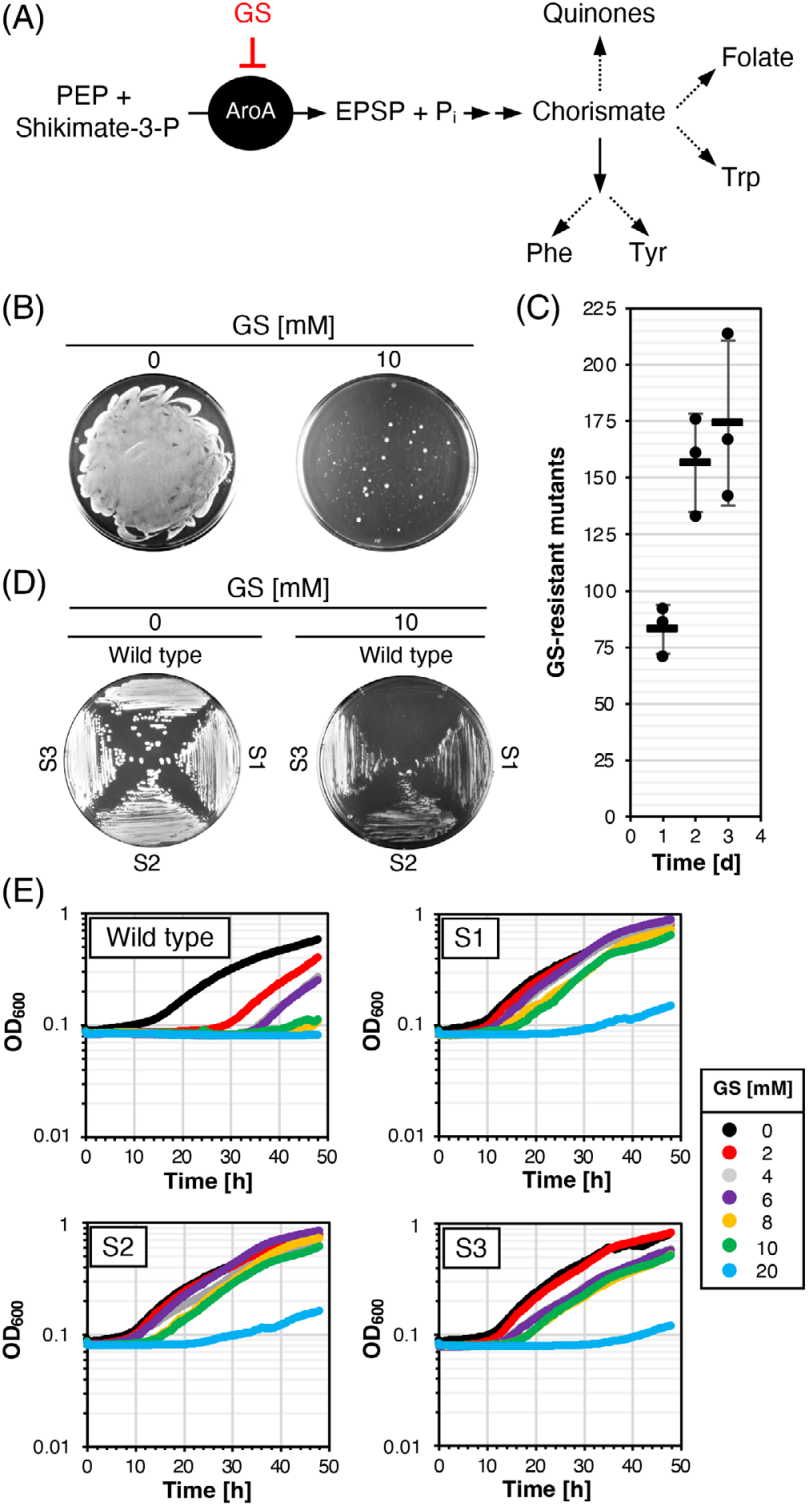
Effect of GS on growth of *Burkholderia anthina*, emergence of GS‐resistant mutants and their characterization. (A) GS inhibits the enolpyruvylshikimate 3‐phosphate (EPSP) synthase AroA. Dashes files indicate that multiple reactions are involved in the biosynthesis of aromatic amino acids, quinones and folates. (B) Emergence of GS‐resistant *B. anthina* mutants. *B. anthina* was grown in C‐Glc medium at 37°C until an OD_600_ of about 2.0. 100 μL containing 10^7^ colony forming units (CFU) were propagated on C‐Glc minimal medium plates that were incubated for 24 h at 37°C. (C) Time‐dependent emergence of GS‐resistant *B. anthina* mutants. *B. anthina* was grown in C‐Glc medium at 37°C until an OD_600_ of about 2.0. 10^7^ CFU were propagated on C‐Glc plates supplemented with 10 mM GS. The plates were incubated for up to 3 days at 37°C and the emerging mutants were counted. Dots indicate biologically independent replicates and bars indicate mean values. (D) Evaluation of growth of the parental strain and three isolated GS‐resistant mutants on C‐Glc plates in the absence and in the presence of the herbicide. The plates were incubated for 24 h at 37°C. (E) Growth of the parental strain and three isolated GS‐resistant mutants in C‐Glc medium supplemented with increasing amounts of GS in a microplate reader at 37°C.

Glyphosate (*N*‐(phosphonomethyl)glycine) (GS) is the most‐commonly used herbicide worldwide (Duke & Powles, 2008; Franz, 1979). GS specifically inhibits the 5‐enolpyruvyl‐shikimate‐3‐phosphate (EPSP) synthase of the shikimate pathway (Figure 1A) (Herrmann & Weaver, 1999; Steinrücken & Amrhein, 1980). The EPSP synthase converts phosphoenolpyruvate (PEP) and shikimate‐3‐phosphate to EPSP, a precursor for chorismate biosynthesis. Chorismate, in turn, is required for de novo synthesis of phenylalanine, tyrosine, and tryptophan as well as of folate and quinones (Herrmann & Weaver, 1999; Steinrücken & Amrhein, 1980). The GS‐dependent inactivation of the EPSP synthase leads to the depletion of the cellular chorismate levels and thus the death of plants and other organisms possessing the EPSP synthase (Fischer et al., 1986; Gresshoff, 1979; Wicke et al., 2019). The effect of GS on EPSP synthase is well studied and understood. GS targets the PEP binding site and competitively inhibits the EPSP synthase (Schönbrunn et al., 2001). The isolation of GS‐insensitive bacterial EPSP synthases and their use to develop herbicide‐tolerant crops allowed weed growth control without harming the growth of the desired plants (Duke & Powles, 2008). The application of GS to fields has put strong selective pressure on microbes and plants to evolve resistance mechanisms against the herbicide (Chekan et al., 2016; Hertel et al., 2021; Wicke et al., 2019). For instance, GS toxicity can be relieved by increasing the copy number of the EPSP synthase gene through selective gene amplification (Gaines et al., 2010; Wicke et al., 2019). The overproduction of the GS‐sensitive EPSP synthase allows the organism to titrate the herbicide and a small portion of the enzyme to function normally. The evolution of GS resistance due to altered transport has also been observed in various organisms including bacteria, fungi, and plants (Ge et al., 2010; Pan et al., 2021; Wicke et al., 2019). For example, plants can become resistant to GS by accumulating mutations resulting in increased vacuolar GS sequestration or by lowering the cytoplasmic GS level (Ge et al., 2010; Pan et al., 2021). Moreover, the degradation of GS by various bacteria is a protective mechanism against the herbicide (Hertel et al., 2021; Hove‐Jensen et al., 2014). GS detoxification by covalent modification also confers resistance against the herbicide (Hertel et al., 2021). For example, the *Escherichia coli* and *Burkholderia pseudomallei* hygromycin phosphotransferases inactivate GS by phosphorylation (Penaloza‐Vazquez et al., 1995; Rao et al., 1983).

In the past years, several GS‐resistant and ‐degrading *Burkholderia* strains have been isolated (Dotson et al., 1996; Hertel et al., 2022; Manogaran, Ahmad, et al., 2018; Manogaran, Shukor, et al., 2018; Penaloza‐Vazquez et al., 1995; Shahid & Khan, 2018). Moreover, it has been observed that the repeated application of GS to soil led to an increase in the abundance of Firmicutes and *Burkholderia* species (Lancaster et al., 2010; Ramirez‐Villacis et al., 2020). Recently, we have identified *Burkholdera anthina* and *Burkholderia cenocepacia* that are resistant to high amounts of GS (Hertel et al., 2022). Here, we evaluated whether GS resistance could be a basic property of the members of the genus *Burkholderia*. For this purpose, we assessed the effect of GS on the *B. anthina* strain DSM 16086. We found that the strain has no intrinsic GS resistance, but quickly develops resistance to the herbicide. Characterization of the evolved strains revealed that GS‐resistance can be acquired by mutations enhancing PEP synthase‐dependent synthesis of PEP, the EPSP synthase co‐substrate that competes with GS for binding to the enzyme. Thus, the overproduction of PEP could be a novel mechanism conferring GS resistance to *Burkholderia* species.

## EXPERIMENTAL PROCEDURES

### Bacterial strains, growth media, culture conditions and chemicals

Bacterial strains are listed in Table S1. The *B. anthina* strain DSM 16086 was obtained from the German Collection of Microorganisms and Cell Cultures GmbH (DSMZ, www.dsmz.de). Chemicals and media were purchased from Sigma–Aldrich (Germany), Carl Roth (Karlsruhe, Germany) and Becton Dickinson (Heidelberg, Germany). *B. subtilis*, *B. anthina* and *E. coli* strains were cultivated in lysogeny broth (LB) and C‐Glc medium. C‐Glc is a minimal medium containing glucose and ammonium as sources of carbon and nitrogen, respectively (Commichau et al., 2007). Pyruvate was used as carbon source as previously described (van den Esker et al., 2017). Agar plates were prepared with 15 g agar/l (Roth, Germany). *E. coli* transformants were selected on LB plates containing kanamycin (50 μg/mL) or ampicillin (100 μg/mL). Growth in liquid medium was monitored using 96‐well plates (Microtest Plate 96‐Well, F Sarstedt, Germany) at 37°C and medium orbital shaking at 237 cpm (4 mm) in a Synergy H1 plate reader (Agilent, USA) equipped with the Gen5 software, and the OD_600_ was measured in 10–15 min intervals. Single colonies were used to inoculate 5 mL overnight LB cultures that were incubated at 220 rpm and 30°C. The OD_600_ was adjusted to 0.1 and 150 μL of the cell suspensions were transferred into 96‐well plates. Bacteria were cultivated in the Synergy H1 plate reader as described above.

### Genome sequencing, assembly, and annotation

Genome sequencing was performed by the G2L (Göttingen, Germany) and GENEWIZ GmbH (Leipzig, Germany). Sequencing libraries were constructed using the Nextera XT DNA sample preparation kit (Illumina, San Diego, CA, USA) and NEB Next Ultra II FS DNA Library Prep 136 (New England Biolabs GmbH, Frankfurt, Germany). Sequencing was realized on a MiSeq instrument using the 2 × 300 bp paired‐end protocol, as recommended by the manufacturer (Illumina, San Diego, CA, USA) and on the NovaSeq6000 using the 2 × 150 bp paired‐end protocol. The quality of the reads was assessed with FastQC version 0.11.9 (Andrews, 2010) and quality was processed using Trimmomatic v.0.36 (Bolger et al., 2014). The Genome of *B. anthina* DSM16086 was assembled with SPAdes version 3.14.0 (Bankevich et al., 2012) with the options—only‐assembler and—isolate. Read coverage was determined with QualiMap v.2.2.1 (Okonechnikov et al., 2016) employing bowtie2 (v.2.2.6) (Langmead & Salzberg, 2012). Contigs < 500 bp and with less than 10% of the average coverage were discarded. The Prokaryotic Genome Annotation Pipeline (PGAP) (Tatusova et al., 2016) was used for automated annotation during genome submission to GenBank. Single‐nucleotide polymorphism (SNP) analyses of the mutant genomes were performed using the breseq pipeline (Deatherage & Barrick, 2014). The annotated draft genome of *B. anthina* DSM16086 is available at GenBank with the accession numbers JAFCIQ010000000.

### DNA manipulation, sequencing, and cloning

Primers were purchased from Sigma–Aldrich (Germany) and are listed in Table S1. Chromosomal DNAs from *B. subtilis*, *B. anthina* and *E. coli* strains were isolated using the peqGOLD Bacterial DNA Kit (Peqlab, Germany). Plasmids were isolated from *E. coli* using the Nucleospin Extract Kit (Macherey and Nagel, Germany). PCR products were purified using the PCR Purification Kit (Qiagen, Germany). Phusion DNA polymerase, restriction enzymes and T4 DNA ligase were purchased from Thermo Scientific (Germany) and used according to the manufacturer's instructions. DNA sequencing was performed at the Microsynth Laboratories (Göttingen, Germany). The *E. coli* strain XL1‐Blue served as the host for plasmid construction. The plasmid pBP1225 for overproduction of N‐terminally Strep‐tagged PpsA from *E. coli* was constructed by generating a PCR fragment using the primer pair IS49/IS50. The PCR product was digested with *Sac*I/*Bam*HI and ligated to pGP172 (Merzbacher et al., 2004) that was cut with the same enzymes. The plasmids pBP1226 and pBP1227 for the overproduction of N‐terminally 6 × His‐tagged PpsR and PpsR Q173P variants, respectively, from *E. coli* were constructed as follows. The *ppsR* wild type allele was amplified with the primer pair IS51/IS52, the PCR product was digested with *Pst*I/*Hind*III and ligated to pWH844 (Schirmer et al., 1997) that was cut with the same enzymes. The *ppsR* A518C allele was generated via the fusion of two PCR products that were generated with the primer pairs IS51/IS63 and IS52/IS64. The resulting *ppsR* A518C allele was introduced via the restriction sites *Pst*I/*Hind*III into the plasmid pWH844. The primer pair IS55/IS56 was used for sequencing the *ppsR* gene in the GS‐resistant *B. anthina* mutants. The plasmid pBP1224 contains the *E. coli ppsA* gene that was amplified with the primer pair IS47/IS48 and ligated to pGP380 that was cut with *Pst*I/*Hind*III (Herzberg et al., 2007).

### Bacterial two‐hybrid (B2H) assay

The primary protein–protein interactions were analysed using the bacterial two‐hybrid (B2H) system that is based on the reconstitution of the adenylate cyclase from *Bordetella pertussis* in the *E. coli cya* mutant BTH101 (Karimova et al., 1998). The plasmid pairs pUT18C/pKT25 and pUT18/pKNT25 were used for the expression of proteins fused to the C and N termini, respectively, of the T18 and T25 fragments of CyaA from *B. pertussis*. The plasmids constructed for the B2H analysis are listed in Table S1. The genes were amplified using the primers listed in Table S1 and cloned between the *Xba*I and *Kpn*I sites of the plasmids pUT18C, pKT25, pUT18 and pKNT25. The *ppsA* and *ppsR*/*ppsR* A518C alleles from *E. coli* DH5α were amplified using the primer pairs IS59/IS60 and IS61/IS62, respectively. The plasmids pBP78–81 and pBP82–pBP85/pBP1228–1231 carry the *E. coli* DH5α *ppsA* and *ppsR*/*ppsR* A518C alleles, respectively (Table S1). The *ppsA* and *ppsR* alleles from the *B. anthina* strain DSM 16086 were amplified using the primer pairs CM1/CM2 and CM3/CM4, respectively. The plasmids pBP1211–pBP1214 and pBP1207–pBP1210 carry the *B. anthina ppsA* and *ppsR* alleles, respectively (Table S1). pUT18C‐zip and pKT25‐zip served as controls. The generated plasmids were used to transform *E. coli* BTH101, and the protein–protein interactions were analysed by plating the cells on LB plates containing 100 μg/mL ampicillin, 50 μg/mL kanamycin, 100 μg/mL X‐Gal and 0.5 mM IPTG. The plates were incubated for 36–72 h at 30°C.

### Protein overproduction and purification

The *E. coli* strains BL21(DE) and DH5α were used for protein overproduction based on the plasmids pGP172 and pWH844, respectively. The strains were cultivated in 1 L LB medium at 37°C. Protein overproduction was induced by the addition of 1 mM IPTG when the cultures had reached an OD_600_ of about 0.6. The cultures were further incubated for 2–3 h at 37°C. The cells were collected by centrifugation and disrupted in lysis buffer (100 mM Tris–HCl pH 8.0, 150 mM NaCl) using a FrenchPress. The soluble protein fraction was separated from the cell debris by centrifugation for 30 min at 10,000 g. The N‐terminally Strep‐tagged PpsA and 6 × His‐tagged PpsR/PpsR Q173P proteins from *E. coli* were purified by Strep‐tag/Streptactin and Ni^2+^‐NTA affinity purification, respectively, as described previously (Commichau et al., 2007; Rosenberg et al., 2015). His‐tagged PpsR Q173P could not be overproduced and purified using the standard procedure. To facilitate the purification of PpsR Q173P, the main culture of the *E. coli* strain DH5α carrying the plasmid pBP1227 was grown in 1 L LB medium supplemented with ampicillin (100 μg/mL) and ethanol (1% (v/v)) at 37°C until an OD_600_ of 0.5. The culture was cooled down, supplemented with 0.1 mM IPTG and incubated overnight at room temperature. After elution, the fractions were tested for the desired protein using 12.5% SDS PAGE gels. Protein concentration was determined using the BioRad dye‐binding assay (BioRad, Germany).

### Metabolomics

The *B. anthina* strains were grown overnight in 4 mL LB medium at 37°C and 160 rpm. The cells were harvested by centrifugation, washed twice in C‐Glc medium, and used to inoculate 100 mL shake flasks containing 10 mL C‐Glc medium to an OD_600_ of 0.1. The cultures were incubated at 37°C and 160 rpm until an OD_600_ of about 0.5. The cultures were further incubated for 30 min, the OD_600_ was determined and the cells in 5 mL of the cultures were passed over a PVDF filter (0.45 μm pore site) in a glass frit. The cells on the filters were resuspended in 1 mL ice‐cold extraction solution (acetonitrile/methanol/ultrapure water, 40%/40%/20%) and incubated for 1 h at −20°C. The cell extracts were centrifuged for 15 min and 20,000 g at −9°C, and stored at −80°C until further processing.

Cell extracts were pooled 1:1 with a ^13^C internal standard as described previously (Guder et al., 2017).

PEP concentrations in cell extracts were measured on an Agilent 6495 triple quadrupole mass quadrupole mass spectrometer equipped with an ESI ion source and coupled to an Agilent 1290 Infinity II UHPLC (both Agilent Technologies) using an iHILIC‐Fusion(P) (50 × 2.1 mm, HILICON AB) column. LC‐solvents were: Solvent A: water with ammonium carbonate (10 mM) and ammonium hydroxide (0.2%). Solvent B: acetonitrile. The LC‐Gradient was: 0 min 90% B, 1.30 min 40% B, 1.5 min 40% B, 1.7  min 90% B, 2.3 min 90% B (flow rate 0.4 mL/min). 3 μL was injected per sample. The settings of the ESI source were: 200°C source gas temperature, 0.4  L/min drying gas and 24 psi nebulizer pressure. The sheath gas temperature was at 300°C and flow at 11 L/min. The electrospray nozzle was set to 500 V and capillary voltage to 2500 V. PEP was analysed in negative ionization mode with a transition from 167 m/z (^12^C) or 170 m/z (^13^C) to 79 (^12^C) or 79 m/z (^13^C) with a collision energy of 29 keV and a dwell time of 12 ms. Raw data were converted into text‐files using MSConvert (Chambers et al., 2012). Data analysis was performed with a customized Matlab script. Relative PEP concentrations are displayed as the ratio between ^12^C and ^13^C signal intensity.

## RESULTS

### Burkholderia anthina *DSM 16086 quickly evolves GS resistance*

Recently, we isolated *B. anthina* and *B. cenocepacia* strains that are highly resistant to GS (Hertel et al., 2022). In contrast to *Bacillus subtilis* and *Escherichia coli* strains that genomically adapted to GS and grew with 10 mM GS, the *Burkholderia* isolates grew in the presence of up to 60 mM GS (Hertel et al., 2022; Wicke et al., 2019). To assess whether it is a general property of *B. anthina* to quickly evolve GS resistance, we propagated the *B. anthina* strain DSM 16086 on C‐Glc minimal agar plates supplemented with 10 mM GS. Pure C‐Glc agar plates served as a control. As shown in Figure 1B, the growth of *B. anthina* was inhibited by 10 mM GS. However, already after 1 day of incubation several GS‐resistant colonies emerged. After 2 days of incubation of the plates, the number of mutants doubled and only a few more mutants emerged after incubation for a total of 3 days (Figure 1C). To verify whether the mutants had acquired stable mutations conferring GS resistance, we randomly isolated three mutants (S1–S3) and performed growth experiment with C‐Glc plates and liquid medium. As shown in Figure 1D,E, the three mutants could grow both on plates and in liquid medium containing 10 mM GS. At a GS concentration of 20 mM, the mutants were no longer able to grow (Figure 1E). To conclude, although the *B. anthina* strain DSM 16086 is not intrinsically resistant to GS, the bacteria quickly develop resistance against GS.

### *Identification of the mutations in the GS‐resistant* B. anthina *mutants*

**FIGURE 2:**
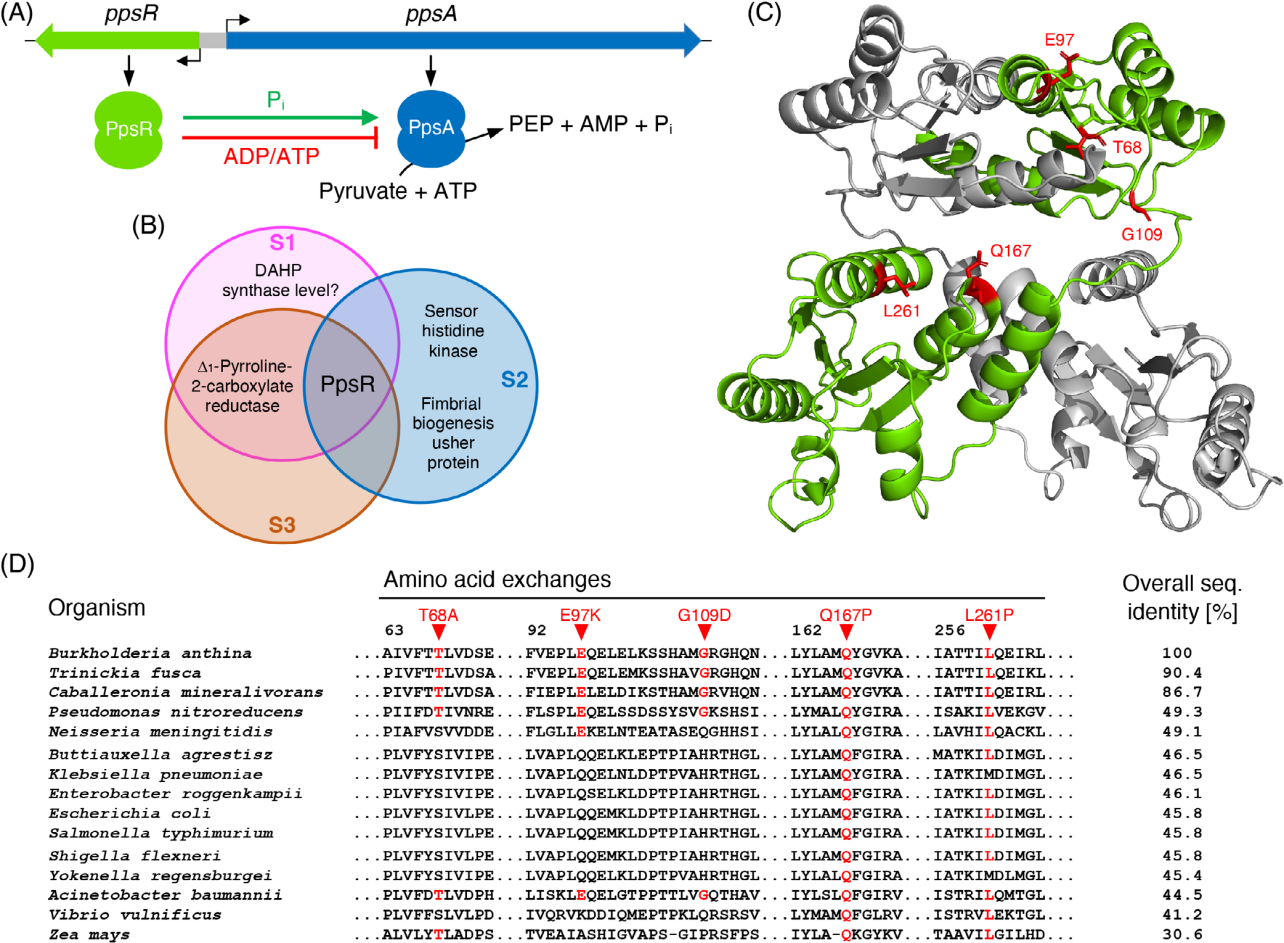
Effect of *ppsR* mutations identified in the GS‐resistant mutants on the cellular PEP level. (A) Schematic illustration of the genomic organization of the *ppsR* and *ppsA* genes and the effect of the PEP synthetase regulatory protein PpsR on the activity of the PEP synthetase PpsA. (B) Venn diagram illustrating the overlap of the genes mutated in *Burkholderia anthina* during growth under selection with GS (see also Table 1). (C) Localization of the amino substitutions that likely affect the function of the PEP synthetase regulatory protein PpsR. The structure model was generated using the SWISS‐MODEL server for homology modelling of protein structures (Waterhouse et al., 2018) and a model of the dimer structure of the maize pyruvate orthophosphate dikinase regulatory protein (PDBid: 5D0N) (Jiang et al., 2016). (D) Sequence alignment of PpsR homologues from *B. anthina* (UniProt code A0A103TDR4), *Trinickia fusca* (UniProt code A0A494XQF5), *Caballeronia mineralivorans* (UniProt code A0A0J1CLS7), *Pseudomonas nitroreducens* (UniProt code A0A246FAH5), *Neisseria menigitidis* (UniProt code Q9K0I1), *Buttiauxella agrestisz* (UniProt code A0A08GFB9), *Klebsiella pneumoniae* (UniProt code B5XQE5), *Enterobacter roggenkampii* (UniProt code A0A167SP00), *Escherichia coli* (UniProt code B1XG10), *Salmonella typhimurium* (UniProt code P67197), *Shigella flexneri* (UniProt code Q0T4R2), *Yokenella regensburgei* (UniProt code A0A6H0K776), *Acinetobacter baumannii* (UniProt code B0V7F2), *Vibrio vulnificus* (UniProt code Q7MF05), and *Zea mays* (UniProt code A0A1D6IDV8). The amino substitutions that likely affect function of the PEP synthetase regulatory protein PpsR are indicated by red triangles.

Genome sequencing analyses of the three GS‐resistant suppressors (S1–S3) revealed that all mutants had acquired mutations in the *ppsR* gene encoding PpsR (Figure 2A,B) (Table 1). In *E. coli*, PpsR regulates the activity of the PEP synthetase PpsA (Burnell, 2010). PpsR activates and inhibits PpsA in a P_i_‐ and ATP/ADP‐dependent manner, respectively (Burnell, 2010). PpsR belongs to the DUF299 protein family that also contains the pyruvate/ortho‐P_i_ dikinase regulatory protein from maize and *Arabidopsis* (Burnell, 2010; Jiang et al., 2016). The suppressors S1 and S3 carry the same mutations in *ppsR* (Table 1). The single nucleotide exchanges in the *ppsR* gene of the suppressors S1/S3 and S2 would cause the amino acids substitutions L261P and Q167P, respectively. Moreover, in the suppressor mutant S1, a 11 bp‐long tandem repeat upstream of the 3‐deoxy‐D‐arabino‐heptulosonate‐7‐phosphate (DAHP) synthase gene was expanded by one repeat unit (Table 1). The DAHP synthase catalyses the conversion of PEP and erythrose‐4‐phosphate to DAHP in the shikimate pathway. It is tempting to speculate that the expansion of the tandem repeat affects the cellular concentration of the DAHP synthase (see Section 3). The suppressors S1 and S3 had also acquired the mutation T886G in Δ_1_‐pyrroline‐2‐carboxylate reductase gene (Table 1). Δ_1_‐Pyrroline‐2‐carboxylate reductase is involved in arginine and proline metabolism and thus not linked to the shikimate pathway (Meister et al., 1957). Furthermore, the suppressor S2 carries single nucleotide exchanges in genes that are predicted to code for a sensor histidine kinase and a fimbria biogenesis protein (Table 1).

**TABLE 1:** Mutations identified in the GS‐resistant *Burkholderia anthina* suppressor mutants.

Strain	Locus tag (gene)	Encoded function	Mutation (position in GenBank sequence)	Effect on gene/protein
Suppressor S1^a^	JQK92_01355 (*ppsR*)	PEP synthetase regulatory protein	T782C	L261P
	JQK92_04345‐50	Upstream of 3‐deoxy‐D‐arabino‐heptulosonate 7‐phosphate synthase	(TACCCGCCCGC)_2→3_ (389344)	Altered expression?
	JQK92_16845	Δ_1_‐Pyrroline‐2‐carboxylate reductase family protein	T886G	S296A
Suppressor S2^a^	JQK92_01355 (*ppsR*)	PEP synthetase regulatory protein	A500C	Q167P
	JQK92_12980	Sensor histidine kinase	G441C	L147L
	JQK92_21945	Fimbrial usher protein	A1039G	N347D
Suppressor S3^a^	JQK92_01355 (*ppsR*)	PEP synthetase regulatory protein	T782C	L261P
	JQK92_16845	Δ_1_‐Pyrroline‐2‐carboxylate reductase family protein	T886G	S296A
Suppressor S4^b^	*ppsR*	PEP synthetase regulatory protein	G289A	E97K
Suppressor S5^b^	*ppsR*	PEP synthetase regulatory protein	G289A	E97K
Suppressor S6^b^	*ppsR*	PEP synthetase regulatory protein	A202G	T68A
Suppressor S7^b^	*ppsR*	PEP synthetase regulatory protein	ΔG282‐A380	C‐terminally truncated by 178 amino acids
Suppressor S8^b^	*ppsR*	PEP synthetase regulatory protein	G326A	G109D
Suppressor S9^b^	*ppsR*	PEP synthetase regulatory protein	A202G	T68A
Suppressor S2.1^a^	JQK92_01355 (*ppsR*)	PEP synthetase regulatory protein	A500C	Q167P
	JQK92_04345‐50	Upstream of 3‐deoxy‐D‐arabino‐heptulosonate 7‐phosphate synthase	(TACCCGCCCGC)_2→3_ (389344)	Altered expression?
	JQK92_12905 (*gpmA*)	2,3‐Diphosphoglycerate‐dependent phosphoglycerate mutase	Δ212GGATGGACC220	Δ71RMD73 Altered enzyme activity?
	JQK92_12980	Sensor histidine kinase	G441C	L147L
	JQK92_16845	Δ_1_‐Pyrroline‐2‐carboxylate reductase family protein	T886G	S296A
	JQK92_21945	Fimbrial usher protein	A1039G	N347D
	JQK92_21215	Upstream of GlxA family transcriptional regulator	G18A (18)	Altered expression?
	JQK92_21965	β‐Ketoacyl‐[acyl‐carrier‐protein] synthase family protein	C19A	Q7K
	JQK92_28130	Sigma 54‐interacting transcriptional regulator	A38T	Q13L
	JQK92_34440	Downstream of DUF488 domain‐containing protein	G55C (55)	Altered expression?
	JQK92_34875	Hypothetical protein	ΔC1‐567 (1–567)	No protein?

^a^ Mutations were identified by genome sequencing.

^b^ Mutations were identified by Sanger sequencing.

Since the *ppsR* gene was mutated in all GS‐resistant suppressor mutants, we assumed that this genomic alteration causes GS resistance in *B. anthina*. Moreover, in *E. coli*, PpsR regulates synthesis of PEP, which is a co‐substrate of the AroA EPSP synthase (Figures 1A and 2A) (Bartlett et al., 2012; Burnell, 2010). Thus, an increased cellular PEP concentration possibly abolishes the GS‐dependent inhibition of the EPSP synthase (see below). Based on the structure of the pyruvate/ortho‐P_i_ dikinase regulatory protein from maize (30.6% overall sequence identify), we generated a structural model for the *B. anthina* PpsR protein (Figure 2C). We also generated a sequence alignment based on sequences of PpsR homologues from bacteria and the maize pyruvate/ortho‐P_i_ dikinase regulatory protein (Figure 2D). As shown in Figure 2C, the residues Q167 and L261 are part of α‐helices that are probably required for the formation of a stable and functional PpsR dimer. Moreover, the sequence alignment revealed that the residues Q167 and L261 are highly conserved among bacterial PpsR homologues and are even present in the maize pyruvate/ortho‐P_i_ dikinase regulator (Figure 2D). Proline has an exceptional conformational rigidity and acts as a structural disruptor in secondary structure elements such as α‐helices and β‐sheets. It is therefore very likely that the Q167P and L261P substitutions in PpsR in the GS‐resistant *B. anthina* suppressors S1–S3 indeed impair the function of the PEP synthetase regulatory protein and thus affect the cellular PEP concentration.

### Glutamine 167 is a critical residue in the PEP synthetase regulatory protein

**FIGURE 3:**
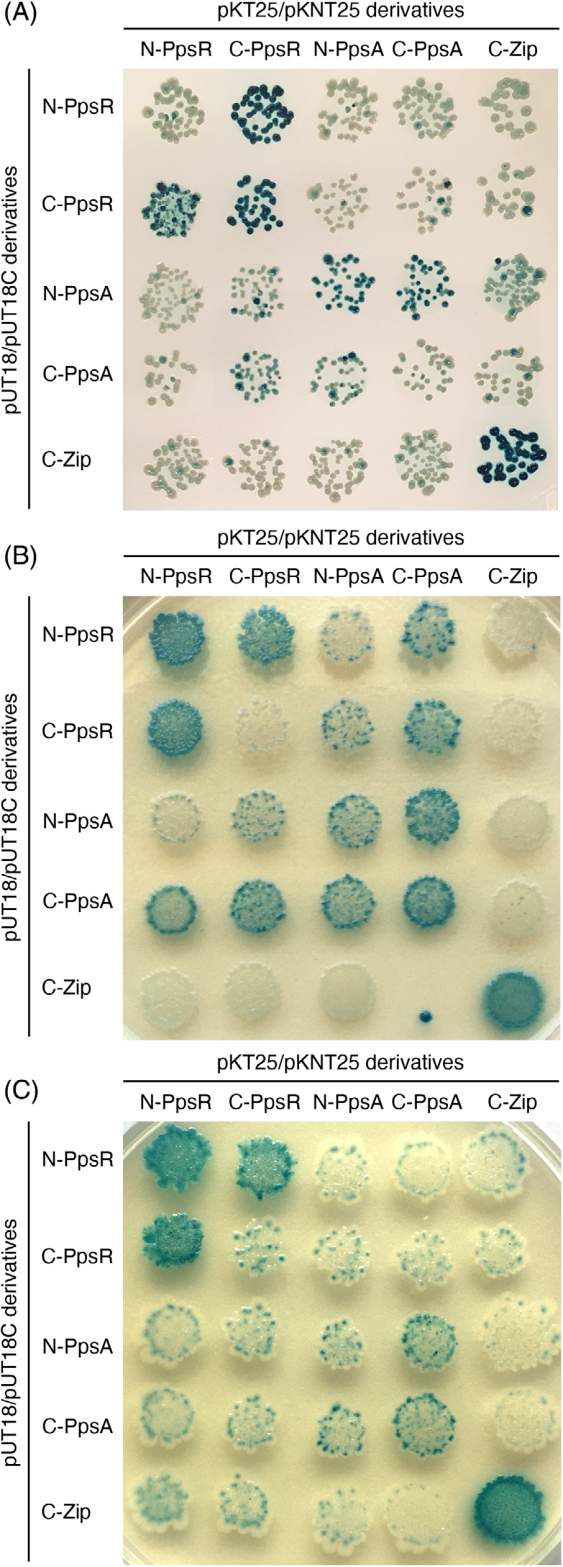
Interaction analysis between PpsR and PpsA homologues from *Burkholderia anthina* and *Escherichia coli*. (A) B2H assay to assess the interaction between PpsR and PpsA from *B. anthina*. (B) B2H assay to assess the interaction between PpsR and PpsA from *E. coli*. The agar plates were incubated for 36 h at 30°C. (C) B2H assay to assess the interaction between PpsR Q173P and PpsA from *E. coli*. The *ppsR* and *ppsA* alleles were introduced into the plasmids pUT18, pUT18C, pKNT25 and pKT25. Plasmids pUT18 and pUT18C allow the expression of the proteins fused to the N‐ and C‐terminus of the T18 domain of the *Bordetella pertussis* adenylate cyclase, respectively. Plasmids pKNT25 and pKT25 allow the expression of the proteins fused to the N‐ and C‐terminus of the T25 domain of the adenylate cyclase. The *E. coli* transformants were spotted onto LB plates supplemented with X‐Gal, IPTG, ampicillin and kanamycin. The agar plate shown in (A) was incubated for 48 h at 30°C, followed by 40 days at 4°C. The agar plates shown in (B) and (C) were incubated for 36 h at 30°C.

Next, we analysed the interaction between PpsR and PpsA from *B. anthina*. For this purpose, we made use of a bacterial two‐hybrid (B2H) system that is based on the functional reconstitution of a split adenylate cyclase from *Bordetella pertussis* in an *E. coli cya* mutant strain (see Section 4). As a control, we analysed the interaction between PpsR and PpsA from *E. coli*. As shown in Figure 3A,B, the PpsR and PpsA homologues from *B. anthina* and *E. coli* showed self‐interaction, which is consistent with previous reports indicating that the proteins form dimers (Jiang et al., 2016; Narindrasorasak & Bridger, 1977). The B2H assay also revealed that *B. anthina* and *E. coli* PpsR and PpsA interact with each other (Figure 3A,B). However, to observe the interaction among the *B. anthina* proteins, the B2H plates indicator strain had to be incubated 25 times longer. Since the genome of *B. anthina* has a GC content of about 66% (https://www.ncbi.nlm.nih.gov/genome/browse/#!/prokaryotes/42805/), the weak interaction between PpsR and PpsA is likely due to the inefficient protein translation.

Next, we aimed to assess the effect of the Q167P substitution on the activity of the PpsR PEP synthases regulatory protein. As shown above, the protein sequence alignment revealed that the residue Q167 is conserved among bacterial PpsR homologues and the maize pyruvate/ortho‐P_i_ dikinase regulator (Figure 2D). Due to the low‐level synthesis of the *B. anthina* proteins in *E. coli*, we thought to study the effect of the Q167P substitution on the activity of the *E. coli* PpsR homologue. For this purpose, we generated plasmids for the overproduction and purification of Strep‐PpsA and His‐PpsR and His‐PpsR Q173P by Strep‐tag/Streptactin‐ and nickel NTA‐affinity purification (see Section 4). The residue Q167 in PpsR from *B. anthina* corresponds to Q173 in *E. coli* PpsR. The purification of the wild type *E. coli* PpsA and PpsR variants was efficient (Figures S1A and S1B). By contrast, we failed to isolate the His‐PpsR Q173P following the standard purification procedure (data not shown). The addition of 1% (v/v) ethanol during the overproduction phase and lowering the cultivation temperature allowed the isolation of small amounts His‐PpsR Q173P. However, His‐PpsR Q173P was not suitable for a PpsA enzyme assay due to contamination with other proteins (Figure S1C). A subsequent B2H analysis to test the PpsA‐PpsR Q173P interaction revealed that the Q173P substitution disturbs protein complex formation, which might also be due to misfolding of PpsR Q173P. To conclude, Q173 (Q167 in *B. anthina* PpsR) seems to be a critical residue for the proper folding and function of the *E. coli* PEP synthetase regulatory protein PpsR.

### *Elevated cellular PEP concentration due to inactivation to PpsR confers* B. anthina *resistance to GS*

**FIGURE 4:**
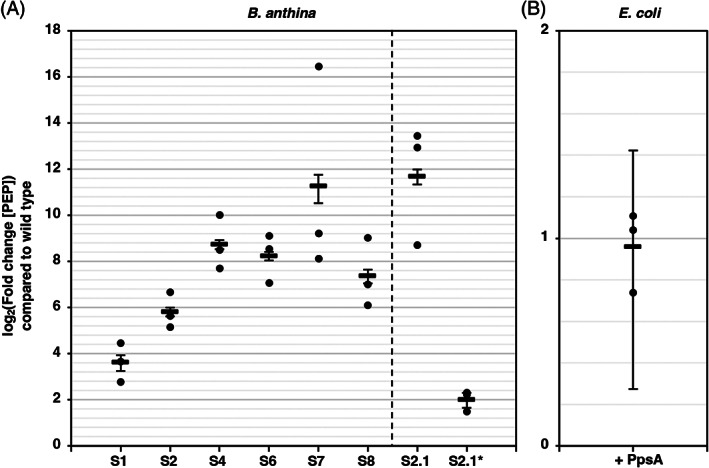
Relative quantification of the cellular PEP concentrations in *Burkholderia anthina* and *Escherichia coli*. (A) Relative quantification of the cellular PEP concentrations in the GS‐resistant *B. anthina* mutants. The asterisk indicates that the PEP concentration was normalized to the parental strain S2.1. (B) Relative quantification of the cellular PEP concentrations *E. coli* strain W3110 expressing the native PpsA enzyme from plasmid pBP1224 (+PpsA). The PEP concentration was normalized to the *E. coli* strain W3110 carrying the empty plasmid pGP380. PEP concentrations in the suppressor mutants are shown as log_2_‐fold changes compared to the wild type concentration. Mean value and standard deviation of three replicates are shown.

To further verify that mutations in the *ppsR* gene confer GS resistance to *B. anthina*, we randomly isolated six additional suppressor mutants (S4–S9) from C‐Glc plates supplemented with 10 mM GS. Sanger sequencing revealed that the mutants had also acquired mutations in the *ppsR* gene (Table 1). The single nucleotide exchanges in the *ppsR* gene of the suppressors S4/S5, S6/S9 and S8 would cause the amino acids substitutions E97K, T68A and G109D, respectively (Figure 2C). The residues T68 and G109 in PpsR are less conserved than the residues at the positions 167 and 261 (Figure 2D). However, the function of the PEP synthetase regulator is certainly affected in the suppressors S4–S8 and S9 because T68, E97 and G109 are substituted by amino acids with different biochemical properties (Table 1). In the GS‐resistant suppressor S7, a 99 bp‐long deletion in the *ppsR* gene leads to a frameshift, which would change the sequence from position 146 and truncate PpsR by 32 amino acids. The loss‐of‐function mutation in *ppsR* of suppressor S7 suggests that the PpsA‐dependent overproduction of PEP likely confers resistance to GS in *B. anthina*. To test this idea, we performed metabolome analyses and determined the relative cellular PEP concentrations in the parental strain and the suppressors S1, S2, S4, S6, S7 and S8 with different amino acid exchanges in PpsR. As shown in Figure 4, the relative amount of PEP was significantly higher in all GS‐resistant suppressor mutants. The variation in PEP levels in the suppressors could be due to the PpsR variants regulating PEP synthetase to different extents. However, the increased synthesis of PEP by PpsA indeed appears to prevent EPSP synthase from being inhibited by GS, thereby allowing the bacteria to grow in the presence of the herbicide.

**FIGURE 5:**
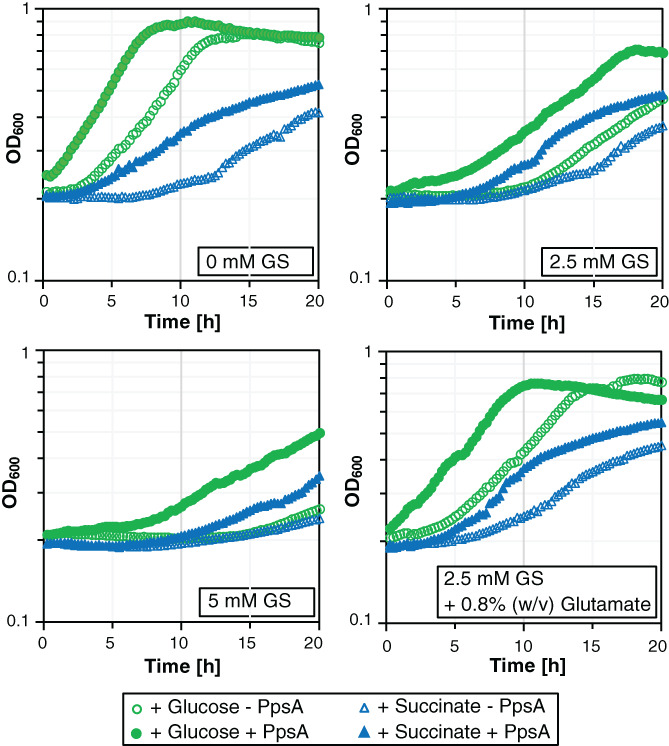
Effect of *ppsA* overexpression on GS resistance in *Escherichia coli*. Growth of the *E. coli* strain W3110 carrying the plasmids pGP380 (−PpsA) and pBP1224 (+PpsA) C‐Glc minimal medium supplemented with the indicated amounts of GS and glutamate in a microplate reader at 37°C.

To assess whether the enhanced synthesis of PEP leads to GS resistance in the *B. subtilis* and *E. coli* strains SP1 and W3110, respectively, we constructed the shuttle vector pBP1224 that carries the *E. coli ppsA* gene. Expression of the *ppsA* gene is driven by the constitutively active *P*_*degQ*_ promoter (Herzberg et al., 2007). Next, the plasmids pGP380 (empty) and pBP1224 (*ppsA*) were introduced into *B. subtilis* and *E. coli*. The expression of the native *ppsA* gene in *E. coli* slightly enhanced growth of the bacteria on C agar plates containing glucose or pyruvate as carbon sources in the absence and in the presence of 2.5 mM GS (Figure S2). By contrast, the overexpression of the *ppsA* gene in *B. subtilis* did not confer GS resistance. Therefore, we only examined the influence of PpsA overexpression on growth and GS resistance in *E. coli* in liquid culture. As shown in Figure 5, the overexpression of PpsA also improved the growth of *E. coli* in C liquid medium containing either glucose or succinate in the absence and in the presence of GS. Thus, the apparently higher GS resistance of *E. coli* is more likely due to a general growth‐promoting effect caused by PpsA. Indeed, metabolome analyses revealed that the relative cellular PEP concentration was not affected by the overexpression of the *ppsA* gene (Figure 4B). The fact that glutamate alleviated the negative effect of GS on the growth of *E. coli* indicates that the growth inhibition was due to the presence of the herbicide that is transported via glutamate uptake systems (Wicke et al., 2019). The unexpected observation that the overexpression of *ppsA* did not increase GS resistance in *B. subtilis* and *E. coli* indicates that the central carbon metabolism is operating in a fundamentally different way in *B. anthina*.

### *A deletion in the phosphoglycerate mutase gene increases GS resistance of a* B. anthina ppsR mutant

**FIGURE 6:**
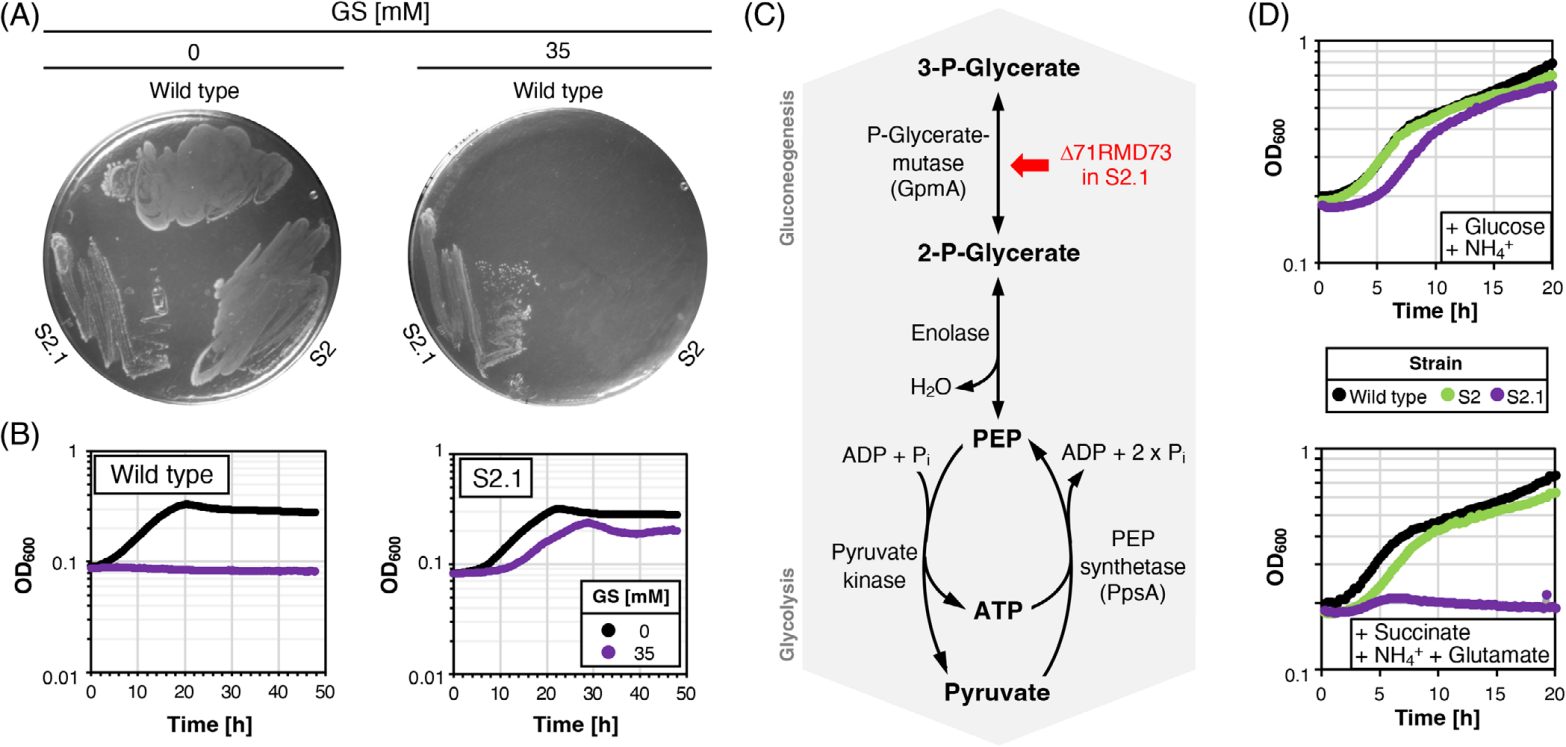
Characterization of the *Burkholderia anthina* mutant with increased GS resistance. (A) Evaluation of growth of the parental strain and isolated GS‐resistant mutant S2.1 on C‐Glc plates in the absence and in the presence of 35 mM GS. The plates were incubated for 24 h at 37°C. (B) Evaluation of growth of the parental strain and isolated GS‐resistant mutant S2.1 in C‐Glc liquid supplemented in the absence and in the presence of 35 mM GS. (C) The pyruvate kinase catalyses the conversion of ADP/Pi and PEP to ATP and pyruvate in the glycolytic pathway. The PEP synthetase converts pyruvate and ATP to AMP/Pi and PEP that is required for gluconeogenesis. (D) Growth of the *B. anthina* mutant S2.1 in C medium supplemented with 0.5% (w/v) glucose, 0.6% (w/v) succinate and 0.8% (w/v) glutamate in a microplate reader at 37°C.

As demonstrated above, the *B. anthina* strain DSM 16086 quickly develops GS resistance by acquiring mutations in the *ppsR* gene (Figure 1B,C). However, the isolated suppressor mutants could not withstand GS concentrations of 20 mM. To assess the potential of a *B. anthina ppsR* mutant to evolve increased GS resistance, we propagated the arbitrarily chosen suppressor S2 on C‐Glc plates supplemented with 35 mM GS. After 3 days of incubation, we identified a single colony, which after its isolation and purification was named suppressor 2.1. As shown in Figure 6A,B, the isolated mutant S2.1 grew on plates and in liquid medium supplemented with 35 mM GS. Genome sequencing analysis revealed that the suppressor S2.1 carries had acquired eight additional mutations in addition to the three already present mutations (Table 1). Like in the suppressor S1, a 11 bp‐long tandem repeat upstream of the 3‐deoxy‐D‐arabino‐heptulosonate‐7‐phosphate (DAHP) synthase gene was expanded by one repeat unit and the Δ_1_‐pyrroline‐2‐carboxylate reductase gene carries the T886G mutation (Table 1). Moreover, the mutations upstream and downstream of *glxA* and a gene encoding the DUF488 protein of unknown function, respectively, probably affect the expression of the respective genes. Other mutations affect the essential β‐ketoacyl‐[acyl‐carrier‐protein] synthase, a sigma 54‐interacting transcriptional regulator and a hypothetical protein (Table 1). Finally, we identified a 9 bp‐long in‐frame deletion in the *gpmA* gene resulting in the loss of three amino acids (Δ71RMD73) in the encoded a P_i_‐glycerate mutase. The P_i_‐glycerate mutase catalyses the reversible conversion of 2‐P_i_‐glycerate to 3‐P_i_‐glycerate in glycolysis and gluconeogenesis (Figure 6C). Growth experiments revealed that the strain S2.1 had lost the ability to grow with the gluconeogenic carbon source succinate (Figure 6D). The deletion of three amino acids in GpmA likely prevents the enzyme from participating in gluconeogenesis. We suspect that the mutation in the *gpmA* gene is responsible for the increased GS resistance of the suppressor S2.1 since an altered activity of the P_i_‐glycerate mutase likely affects the PEP pool (Figure 6C). Indeed, metabolome analyses revealed that the relative cellular PEP concentration was increased as compared to the parental strain S2 (Figure 4A). To conclude, a further increase in the cellular PEP level by altered GpmA activity prevents EPSP synthase from being inhibited by elevated GS concentrations (see Section 3).

## DISCUSSION

**FIGURE 7:**
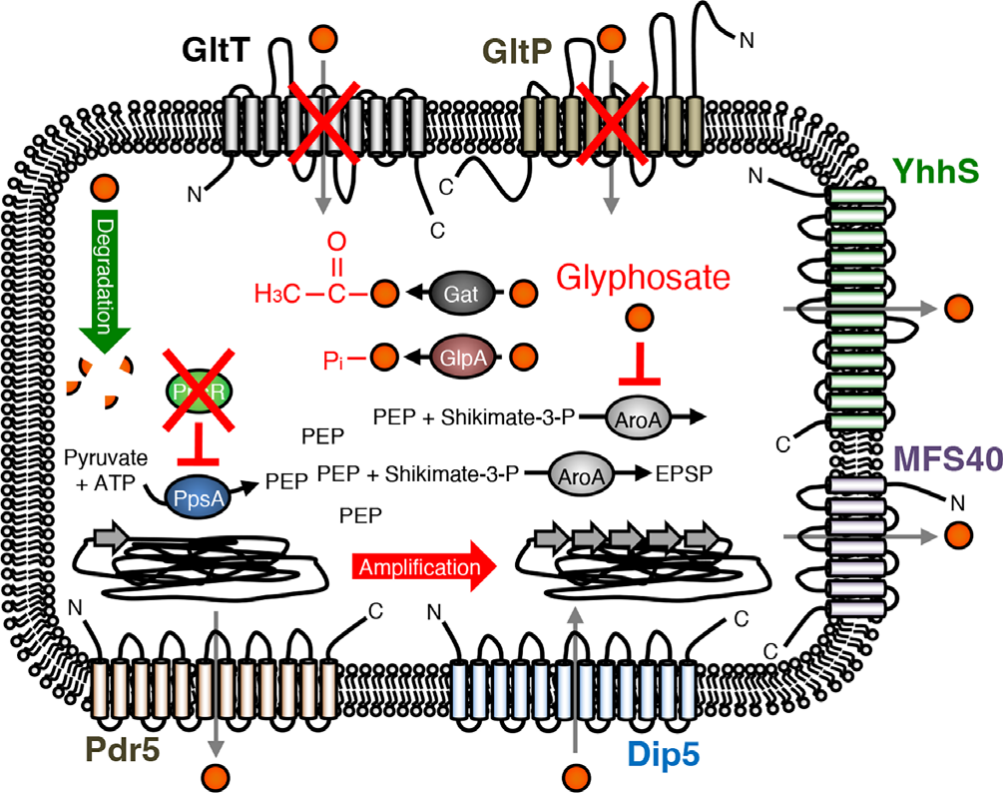
Mechanisms conferring GS resistance and enzymes that catalyse pyruvate and PEP synthesis. Mechanisms conferring GS resistance in bacteria and fungi. AroA, EPSP synthase; GltT and GltP, *Bacillus subtilis* glutamate transporters; YhhS and MFS40, major facilitator secondary transporters from *Escherichia coli* and *Aspergillus oryzae*, respectively; Pdr5 and Dip5 are amino acid and ABC efflux transporters, respectively, from *Saccharomyces cerevisiae*; Gat, *Bacillus licheniformis* GS *N‐*acetyltransferase; GlpA, *Burkholderia pseudomallei* hygromycin phosphotransferase; PpsA, PEP synthetase; PpsR, PEP synthetase regulatory protein; PEP, phosphoenolpyruvate; S3P, shikimate‐3‐phosphate; EPSP, enolpyruvylshikimate‐3‐phosphate.

Unlike the previously characterized *B. anthina* and *B. cenocepacia* isolates that grew in the presence of 60 mM GS (Hertel et al., 2021), the *B. anthina* strain DSM 16086 shows no intrinsic GS resistance. However, *B. anthina* DSM 16086 quickly acquires resistance to GS during growth on minimal medium agar plates supplemented with 10 mM GS. Sequencing analyses revealed that all GS‐resistant *B. anthina* mutants had acquired mutations in the *ppsR* gene encoding PpsR, which shares 45.8% overall sequence identity with the homologue of *E. coli* (Figure 2D). In *E. coli* it has been demonstrated that PpsR regulates the activity of PpsA in a P_i_‐ and ATP/ADP‐dependent manner (Bartlett et al., 2012; Burnell, 2010). The PEP synthetase PpsA is a gluconeogenic enzyme that is required for the growth of *E. coli* on three‐carbon substrates like pyruvate (Antonovsky et al., 2016; Cooper & Kornberg, 1965, 1967; Cooper & Kornberg, 1969; Herz et al., 2017; Niersbach et al., 1992). Metabolome analysis revealed that the cellular concentration of PEP, the co‐substrate of the EPSP synthase of the shikimate pathway, was elevated in the *B. anthina* GS‐resistant mutants. Since GS competes with PEP for binding to the EPSP synthase, we suspect that the increased cellular concentration of PEP due to the mutational inactivation of the *ppsR* gene allows the *B. anthina* mutants to grow in the presence of GS (Figure 7). A previous study also identified residues in the *E. coli* PpsR protein that are critical for enzyme folding and catalysis (Bartlett et al., 2012). However, here, we identified different amino acid residues that are crucial for proper enzyme function. It is interesting to note that despite the presence of a futile cycle that involves the ATP‐generating pyruvate kinase and the ATP‐consuming PEP synthetase the de‐regulation resulted in a significant increase in the cellular PEP concentration in the GS‐resistant *B. anthina* mutants (Figure 6C). In *E. coli*, it has indeed been shown that the overexpression of the *ppsA* gene stimulates oxygen and sugar consumption (Patnaik et al., 1992). However, we observed that the overexpression of the *E. coli ppsA* gene in *B. subtilis* and *E. coli* did not confer GS resistance. Recently, it has been shown that the overexpression of the native *ppsA* gene only slightly improved metabolites of the PEP‐consuming shikimate pathway (Chen et al., 2014). Thus, the pyruvate kinase activity probably prevents the accumulation of PEP in *E. coli* and probably also in *B. subtilis*. This unexpected result might be due to fundamental differences in the regulation of central carbon metabolism in *B. anthina* as compared *B. subtilis* and *E. coli*. Thus, the genomic adaptation uncovered a novel mechanism conferring GS resistance that is probably unique to *B. anthina*. In the present study, we also found that further adaptation of *B. anthina* resulted in the acquisition of a mutation in the *gpmA* gene encoding the P_i_‐glycerate mutase that catalyses the conversion of 2‐P_i_‐glycerate to 3‐P_i_‐glycerate in glycolysis and gluconeogenesis (Figure 6C). The altered activity of the P_i_‐glycerate mutase also affects the PEP pool, thereby preventing the inhibition of the EPSP synthase by elevated GS concentrations.

Previously, it has been shown that also *B. subtilis* and *E. coli* rapidly adapt to GS at the genome level (Wicke et al., 2019). In *B. subtilis*, the mutational inactivation of the *gltT* and *gltP* genes, encoding the promiscuous high‐ and low‐affinity glutamate transporters GltT and GltP, respectively, confers resistance against GS as well as against glufosinate that inhibits glutamine biosynthesis (Wicke et al., 2019) (Figure 7). In *E. coli* mutations that affect the activity or the cellular concentration of the EPSP synthase by an amino acid substitution in the GS target and by promoter‐up as well as selective gene amplification, respectively, allow the bacteria to grow in the presence of 10 mM GS (Figure 7) (Wicke et al., 2019). Moreover, altered transport of GS via the transporter proteins was also associated with variations in GS resistance in fungi and plants (Figure 7) (Pan et al., 2021; Staub et al., 2012; Tao et al., 2017). Furthermore, bacteria can detoxify GS by covalent modification and degrade the herbicide (Figure 7) (Hertel et al., 2021; Hove‐Jensen et al., 2014; Penaloza‐Vazquez et al., 1995; Rao et al., 1983). Thus, the isolation and characterization of GS‐resistant bacteria revealed various mechanisms conferring GS resistance.

## AUTHOR CONTRIBUTIONS

**Inge Schwedt:** Formal analysis (lead); investigation (lead); methodology (lead); supervision (equal). **Madeline Collignon:** Investigation (equal). **Carolin Mittelstädt:** Investigation (supporting). **Florian Giudici:** Investigation (supporting). **Johanna Rapp:** Investigation (supporting); methodology (supporting). **Janek Meißner:** Investigation (supporting). **Hannes Link:** Investigation (supporting); methodology (supporting). **Robert Hertel:** Data curation (supporting); formal analysis (supporting); investigation (supporting). **Fabian M. Commichau:** Conceptualization (lead); formal analysis (lead); project administration (lead).

## CONFLICT OF INTEREST STATEMENT

The authors certify that there is no potential conflict of interest.

## Supporting information


**Table S1.** Bacterial strains, primers, and plasmids.
**Figure S1.** Purification of *E. coli* PpsA, PpsR and PpsR Q173P.
**Figure S2.** Effect of *ppsA* overexpression on GS resistance in *B. subtilis* and *E. coli*.
Supporting information references.

## Data Availability

The authors confirm that the data supporting the findings of this study are available within the article and its supplementary materials.

## References

[emi413184-bib-0001] Andrews, S. (2010) FastQC: a quality control tool for high throughput sequence data . Available from: https://www.bioinformatics.babraham.ac.uk/projects/fastqc/

[emi413184-bib-0002] Antonovsky, N. , Gleizer, S. , Noor, E. , Zohar, Y. , Herz, E. , Barenholz, U. et al. (2016) Sugar synthesis from CO_2_ in *Escherichia coli* . Cell, 166, 115–125. 27345370 10.1016/j.cell.2016.05.064 PMC4930481

[emi413184-bib-0003] Bankevich, A. , Nurk, S. , Antipov, D. , Gurevich, A.A. , Dvorkin, M. , Kulikov, A.S. et al. (2012) SPAdes: a new genome assembly algorithm and its applications to single‐cell sequencing. Journal of Computational Biology, 19, 455–477. 22506599 10.1089/cmb.2012.0021 PMC3342519

[emi413184-bib-0004] Bartlett, S. , Seeliger, J. & Burnell, J.N. (2012) Identification of critical residues in the bifunctional phosphoenolpyruvate synthetase kinase/phosphotransferase of *Escherichia coli* . Current Topics in Biochemical Research, 14, 77–83.

[emi413184-bib-0005] Bolger, A.M. , Lohse, M. & Usadel, B. (2014) Trimmomatic: a flexible trimmer for Illumina sequence data. Bioinformatics, 30, 2114–2120. 24695404 10.1093/bioinformatics/btu170 PMC4103590

[emi413184-bib-0006] Burnell, J.N. (2010) Cloning and characterization of *Escherichia coli* DUF299: a bifunctional ADP‐dependent kinase—Pi‐dependent pyrophosphorlyase from bacteria. BMC Biochemistry, 11, 1. 20044937 10.1186/1471-2091-11-1 PMC2817694

[emi413184-bib-0007] Chambers, M.C. , Maclean, B. , Burke, R. , Amodei, D. , Ruderman, D.L. , Neumann, S. et al. (2012) A cross‐platform toolkit for mass spectrometry and proteomics. Nature Biotechnology, 30, 918–920. 10.1038/nbt.2377 PMC3471674 23051804

[emi413184-bib-0008] Chekan, J.R. , Cogan, D.P. & Nair, S.K. (2016) Molecular basis for resistance against phosphonate antibiotics and herbicides. MedChemComm, 7, 28–36. 26811741 10.1039/C5MD00351B PMC4723106

[emi413184-bib-0009] Chen, X. , Li, M. , Zhou, L. , Shen, W. , Algasan, G. , Fan, Y. et al. (2014) Metabolic engineering of *Escherichia coli* for improving shikimate synthesis from glucose. Bioresource Technology, 166, 64–71. 24905044 10.1016/j.biortech.2014.05.035

[emi413184-bib-0010] Commichau, F.M. , Herzberg, C. , Tripal, P. , Valerius, O. & Stülke, J. (2007) A regulatory protein‐protein interaction governs glutamate biosynthesis in *Bacillus subtilis*: the glutamate dehydrogenase RocG moonlights in controlling the transcription factor GltC. Molecular Microbiology, 65, 642–654. 17608797 10.1111/j.1365-2958.2007.05816.x

[emi413184-bib-0011] Cooper, R.A. & Kornberg, H.L. (1965) Net formation of phosphoenolpyruvate from pyruvate by *Escherichia coli* . Biochimica et Biophysica Acta, 104, 618–620. 5322808 10.1016/0304-4165(65)90374-0

[emi413184-bib-0012] Cooper, R.A. & Kornberg, H.L. (1967) The direct synthesis of phosphoenolpyruvate from pyruvate by *Escherichia coli* . Proceedings of the Royal Society of London: Series B: Biological Sciences, 168, 263–289. 4383554 10.1098/rspb.1967.0065

[emi413184-bib-0013] Cooper, R.A. & Kornberg, H.L. (1969) Phosphoenolpyruvate synthetase. Methods in Enzymology, 13, 309–314.

[emi413184-bib-0014] Deatherage, D.E. & Barrick, J.E. (2014) Identification of mutations in laboratory‐evolved microbes from next‐generation sequencing data using breseq. Methods in Molecular Biology, 1151, 165–188. 24838886 10.1007/978-1-4939-0554-6_12 PMC4239701

[emi413184-bib-0015] Dotson, S.B. , Smith, C.E. , Ling, C.S. , Barry, G.F. & Kishore, G.M. (1996) Identification, characterization, and cloning of a phosphonate monoester hydrolase from *Burkholderia caryophilli* PG2982. The Journal of Biological Chemistry, 271, 25754–25761. 8824203 10.1074/jbc.271.42.25754

[emi413184-bib-0016] Duke, S.O. & Powles, S.B. (2008) Glyphosate: a once‐in‐a‐century herbicide. Pest Management Science, 64, 319–325. 18273882 10.1002/ps.1518

[emi413184-bib-0017] Fischer, R.S. , Berry, A. , Gaines, C.G. & Jensen, R.A. (1986) Comparative action of glyphosate as a trigger of energy drain in eubacteria. Journal of Bacteriology, 168, 1147–1154. 3096971 10.1128/jb.168.3.1147-1154.1986 PMC213615

[emi413184-bib-0018] Franz, J.E. (1979) In: Geissbuehler, H. (Ed.) Advances in Pesticide Science, Vol. 2. Oxford and New York: Pergamon Press, pp. 139–147.

[emi413184-bib-0019] Gaines, T.A. , Zhang, W. , Wang, D. , Bukun, B. , Chisholm, S.T. , Shaner, D.L. et al. (2010) Gene amplification confers glyphosate resistance in *Amaranthus palmeri* . Proceedings of the National Academy of Sciences of the United States of America, 107, 1029–1034. 20018685 10.1073/pnas.0906649107 PMC2824275

[emi413184-bib-0020] Ge, X. , André d'Avignon, D. , Ackerman, J.J.H. & Sammons, R.D. (2010) Rapid vacuolar sequestration: the horseweed glyphosate resistance mechanism. Pest Management Science, 66, 345–348. 20063320 10.1002/ps.1911 PMC3080097

[emi413184-bib-0021] Gresshoff, P.M. (1979) Growth inhibition by glyphosate and reversal of its actions by phenylalanine and tyrosine. Australian Journal of Plant Physiology, 6, 177–185.

[emi413184-bib-0022] Guder, C.J. , Schramm, T. , Sander, T. & Link, H. (2017) Time‐optimized isotope ratio LC‐MS/MS for high‐throughput quantification of primary metabolites. Analytical Chemistry, 89, 1624–1631. 28050903 10.1021/acs.analchem.6b03731

[emi413184-bib-0023] Herrmann, K.M. & Weaver, L.M. (1999) The shikimate pathway. Annual Review of Plant Physiology and Plant Molecular Biology, 78, 176–197. 10.1146/annurev.arplant.50.1.473 15012217

[emi413184-bib-0024] Hertel, R. , Gibhardt, J. , Martienssen, M. , Kuhn, R. & Commichau, F.M. (2021) Molecular mechanisms underlying glyphosate resistance in bacteria. Environmental Microbiology, 23, 2891–2905. 33876549 10.1111/1462-2920.15534

[emi413184-bib-0025] Hertel, R. , Schöne, K. , Mittelstädt, C. , Meißner, J. , Zschoche, N. , Collignon, M. et al. (2022) Characterization of glyphosate‐resistant *Burkholderia anthina* and *Burkholderia cenocepacia* isolates from a commercial Roundup® solution. Environmental Microbiology Reports, 14, 70–84. 34786867 10.1111/1758-2229.13022

[emi413184-bib-0026] Herz, E. , Antonovsky, N. , Bar‐On, Y. , Davidi, D. , Gleizer, S. , Prywes, N. et al. (2017) The genetic basis for the adaptation of *E. coli* to sugar synthesis from CO_2_ . Nature Communications, 8, 1705. 10.1038/s41467-017-01835-3 PMC5700066 29167457

[emi413184-bib-0027] Herzberg, C. , Weidinger, L.A.F. , Dörrbecker, B. , Hübner, S. , Stülke, J. & Commichau, F.M. (2007) SPINE: a method for the rapid detection and analysis of protein‐protein interactions *in vivo* . Proteomics, 7, 4032–4035. 17994626 10.1002/pmic.200700491

[emi413184-bib-0028] Hove‐Jensen, B. , Zechel, D.L. & Jochimsen, B. (2014) Utilization of glyphosate as phosphate source: biochemistry and genetics of bacterial carbon lyase. Microbiology and Molecular Biology Reviews, 78, 176–197. 24600043 10.1128/MMBR.00040-13 PMC3957732

[emi413184-bib-0029] Jiang, L. , Chen, Y.B. , Zheng, J. , Chen, Z. , Liu, Y. , Tao, Y. et al. (2016) Structural basis of reversible phosphorylation by maize pyruvate orthophosphate dikinase regulatory protein. Plant Physiology, 170, 732–741. 26620526 10.1104/pp.15.01709 PMC4734583

[emi413184-bib-0030] Karimova, G. , Pidoux, J. , Ullmann, A. & Landant, D. (1998) A bacterial two‐hybrid system based on a reconstituted signal transduction pathway. Proceedings of the National Academy of Sciences of the United States of America, 95, 5752–5756. 9576956 10.1073/pnas.95.10.5752 PMC20451

[emi413184-bib-0031] Lancaster, S.H. , Hollister, E.B. , Senseman, S.A. & Gentry, T.J. (2010) Effects of repeated glyphosate applications on soil microbial community composition and the mineralization of glyphosate. Pest Management Science, 66, 59–64. 19697445 10.1002/ps.1831

[emi413184-bib-0032] Langmead, B. & Salzberg, S.L. (2012) Fast gapped‐read alignment with Bowtie 2. Nature Methods, 9, 357–359. 22388286 10.1038/nmeth.1923 PMC3322381

[emi413184-bib-0033] Manogaran, M. , Ahmad, S.A. , Yasid, N.A. , Yakasai, H.M. & Shukor, M.Y. (2018) Characterization of the simultaneous molybdenum reduction and glyphosate degradation by *Burkholderia vietnamiensis* AQ5‐12 and *Burkholderia* sp. AQ5‐13. 3 Biotech, 8, 117. 10.1007/s13205-018-1141-2 PMC5801107 29430378

[emi413184-bib-0034] Manogaran, M. , Shukor, M.Y. , Yasid, N.A. , Khalil, K.A. & Ahmad, S.A. (2018) Optimisation of culture composition for glyphosate degradation by *Burkholderia vietnamiensis* strain AQ5‐12. 3‐Biotech, 8, 108. 29430369 10.1007/s13205-018-1123-4 PMC5794677

[emi413184-bib-0035] Meister, A. , Radhakrishnan, A.N. & Buckley, S.D. (1957) Enzymatic synthesis of L‐pipecolic acid and L‐proline. The Journal of Biological Chemistry, 229, 789–800. 13502341

[emi413184-bib-0036] Merzbacher, M. , Detsch, C. , Hillen, W. & Stülke, J. (2004) *Mycoplasma pneumoniae* HPr kinase/phosphorylase. European Journal of Biochemistry, 271, 367–374. 14717704 10.1046/j.1432-1033.2003.03935.x

[emi413184-bib-0037] Narindrasorasak, S. & Bridger, W.A. (1977) Phosphenolpyruvate synthetase of *Escherichia coli*: molecular weight, subunit composition, and identification of phosphohistidine in phosphoenzyme intermediate. The Journal of Biological Chemistry, 252, 3121–3127. 16880

[emi413184-bib-0038] Niersbach, M. , Kreuzaler, F. , Geerse, R.H. , Postma, P.W. & Hirsch, H.J. (1992) Cloning and nucleotide sequencing of the *Escherichia coli* K‐12 *ppsA* gene, encoding PEP synthase. Molecular & General Genetics, 231, 332–336. 1310524 10.1007/BF00279808

[emi413184-bib-0039] Okonechnikov, K. , Conesa, A. & García‐Alcalde, F. (2016) Qualimap 2: advanced multi‐sample quality control for high‐throughput sequencing data. Bioinformatics, 32, 292–294. 26428292 10.1093/bioinformatics/btv566 PMC4708105

[emi413184-bib-0040] Pan, L. , Yu, Q. , Wang, J. , Han, H. , Mao, L. , Nyporko, A. et al. (2021) An ABCC‐type transporter endowing glyphosate resistance in plants. Proceedings of the National Academy of Sciences of the United States of America, 118, e2100136118. 33846264 10.1073/pnas.2100136118 PMC8072331

[emi413184-bib-0041] Patnaik, R. , Roof, W.D. , Young, R.F. & Liao, J.C. (1992) Stimulation of glucose catabolism in *Escherichia coli* by a potential futile cycle. Journal of Bacteriology, 174, 7527–7532. 1332936 10.1128/jb.174.23.7527-7532.1992 PMC207462

[emi413184-bib-0042] Penaloza‐Vazquez, A. , Mena, G.L. , Herrera‐Estrella, L. & Bailey, A.M. (1995) Cloning and sequencing of the genes involved in glyphosate utilization by *Pseudomonas pseudomallei* . Applied and Environmental Microbiology, 61, 538–543. 7574593 10.1128/aem.61.2.538-543.1995 PMC167315

[emi413184-bib-0043] Ramirez‐Villacis, D.X. , Finkel, O.M. , Salas‐González, I. , Fitzpatrick, C.R. , Dangl, J.L. , Jones, C.D. et al. (2020) Root microbiome modulates plant growth promotion induced by low doses of glyphosate. mSphere, 5, e00484–e00420. 32817451 10.1128/mSphere.00484-20 PMC7426167

[emi413184-bib-0044] Rao, R.N. , Allen, N.E. , Hobbs, J.N. , Alborn, W.E. , Kirst, H.A. & Paschal, J.W. (1983) Genetic and enzymatic basis of hygromycin B resistance in *Escherichia coli* . Antimicrobial Agents and Chemotherapy, 24, 689–695. 6318654 10.1128/aac.24.5.689 PMC185926

[emi413184-bib-0045] Rosenberg, J. , Dickmanns, A. , Neumann, P. , Gunka, K. , Arens, J. , Kaever, V. et al. (2015) Structural and biochemical analysis of the essential diadenylate cyclase CdaA from *Listeria monocytogenes* . The Journal of Biological Chemistry, 290, 6596–6606. 25605729 10.1074/jbc.M114.630418 PMC4358292

[emi413184-bib-0046] Schirmer, F. , Ehrt, S. & Hillen, W. (1997) Expression, inducer spectrum, domain structure, and function of MopR, the regulator of phenol degradation in *Acinetobacter calcoaceticus* NCIB8250. Journal of Bacteriology, 179, 1329–1336. 9023219 10.1128/jb.179.4.1329-1336.1997 PMC178833

[emi413184-bib-0047] Schönbrunn, H.C. , Eschenburg, S. , Shuttleworth, W.A. , Schloss, J.V. , Amrhein, N. , Evans, J.N. et al. (2001) Interaction of the herbicide glyphosate with its target enzyme 5‐enolpyruvylshikimate 3‐phosphate synthase in atomic detail. Proceedings of the National Academy of Sciences of the United States of America, 98, 1376–1380. 11171958 10.1073/pnas.98.4.1376 PMC29264

[emi413184-bib-0048] Shahid, M. & Khan, M.S. (2018) Glyphosate induced toxicity to chickpea plants and stress alleviation by herbicide tolerant phosphate solubilizing *Burkholderia cepacia* PSBB1 carrying multifarious plant growth promoting activities. 3 Biotech, 8, 131. 10.1007/s13205-018-1145-y PMC5812922 29450121

[emi413184-bib-0049] Staub, J.M. , Brand, L. , Tran, M. , Kong, Y. & Rogers, S.G. (2012) Bacterial glyphosate resistance conferred by overexpression of an *E. coli* membrane efflux transporter. Journal of Industrial Microbiology & Biotechnology, 39, 641–647. 22089966 10.1007/s10295-011-1057-x

[emi413184-bib-0050] Steinrücken, H.C. & Amrhein, N. (1980) The herbicide glyphosate is a potent inhibitor of 5‐enolpyruvyl‐shikimic acid‐3‐phosphate synthase. Biochemical and Biophysical Research Communications, 94, 1207–1212. 7396959 10.1016/0006-291x(80)90547-1

[emi413184-bib-0051] Tao, B. , Shao, B.H. , Qiao, Y.X. , Wang, X.Q. , Chang, S.J. & Qiu, L.J. (2017) Identification and functional analysis of a new glyphosate resistance gene from a fungus cDNA library. Pesticide Biochemistry and Physiology, 140, 65–68. 28755696 10.1016/j.pestbp.2017.05.013

[emi413184-bib-0052] Tatusova, T. , DiCuccio, M. , Badretdin, A. , Chetvernin, V. , Nawrocki, E.P. , Zaslavsky, L. et al. (2016) NCBI prokaryotic genome annotation pipeline. Nucleic Acids Research, 44, 6614–6624. 27342282 10.1093/nar/gkw569 PMC5001611

[emi413184-bib-0053] van den Esker, M.H. , Kovács, A.T. & Kuipers, O.P. (2017) YsbA and LytST are essential for pyruvate utilization in *Bacillus subtilis* . Environmental Microbiology, 19, 83–94. 27422364 10.1111/1462-2920.13454

[emi413184-bib-0054] Waterhouse, A. , Bertoni, M. , Bienert, S. , Studer, G. , Tauriello, G. , Gumienny, R. et al. (2018) SWISS‐MODEL: homology modelling of protein structures and complexes. Nucleic Acids Research, 46, W296–W303. 29788355 10.1093/nar/gky427 PMC6030848

[emi413184-bib-0055] Wicke, D. , Schulz, L.M. , Lentes, S. , Scholz, P. , Poehlein, A. , Gibhardt, J. et al. (2019) Identification of the first glyphosate transporter by genomic adaptation. Environmental Microbiology, 21, 1287–1305. 30666812 10.1111/1462-2920.14534

