# Supplementary material for: Genomic adaptation of Burkholderia anthina to glyphosate uncovers a novel herbicide resistance mechanism
Source: Environ Microbiol Rep. 2023 Jun 13;15(6):727–39. doi: 10.1111/1758-2229.13184 (PMC10667639; doi:10.1111/1758-2229.13184)
Supplement: Supplementary file 1 — Table S1. Bacterial strains, primers, and plasmids. Figure S1. Purification of E. coli PpsA, PpsR and PpsR Q173P. Figure S2. Effect of ppsA overexpression on GS resistance in B. subtilis and E. coli. Supporting information references. [file EMI4-15-727-s001.docx]

**Genomic adaptation of *Burkholderia anthina* to glyphosate uncovers**

**a novel herbicide resistance mechanism**

**Inge Schwedt^1,2^, Madeline Collignon^1^, Carolin Mittelstädt^1^, Florian Giudici^1^, Johanna Rapp^3^, Janek Meißner^4^, Hannes Link^3^, Robert Hertel^1,5^, Fabian M. Commichau^1,2^***

^1^FG Synthetic Microbiology, Institute for Biotechnology, BTU Cottbus-Senftenberg, Senftenberg, Germany

^2^FG Molecular Microbiology, Institute of Biology, University of Hohenheim, Stuttgart, Germany

^3^Interfaculty Institute for Microbiology and Infection Medicine Tübingen, University of Tübingen, Bacterial Metabolomics, Tübingen, Germany

^4^Department of General Microbiology, Institute for Microbiology and Genetics, University of Goettingen, Göttingen, Germany

^5^Department of Genomic and Applied Microbiology, Institute for Microbiology and Genetics, University of Goettingen, Göttingen, Germany

*Correspondence to Fabian Commichau, fabian.commichau@uni-hohenheim.de

Universität Hohenheim

FG Molecular Microbiology

Institute for Biology

70599 Stuttgart

Phone: +49-(0)711-459-22222; Fax: +49-(0)711-459-22238

Mail: fabian.commichau@uni-hohenheim.de

**Supporting information**

**Table S1.** Bacterial strains, primers and plasmids.

**Fig. S1.** Purification of *E. coli* PpsA, PpsR and PpsR Q173P.

**Fig. S2.** Effect of *ppsA* overexpression on GS resistance in *B. subtilis* and *E. coli.*

**Supporting information references**

**Table S1: Bacterial strains, primers and plasmids**

| **Bacterial strains** | **Genotype; construction** | **Reference** |
| --- | --- | --- |
| *Bacillus subtilis* SP1 | **-** | Richts et al., 2020; Laboratory strain collection |
| *Burkholderia anthina* DSM 16086 | **-** | [www.dsmz.de](http://www.dsmz.de); Vandamme et al., 2002 |
| *B. anthina* S1 | Glyphosate-resistant suppressor, derived from *B anthina* DSM 16086 | This study |
| *B. anthina* S2 | Glyphosate-resistant suppressor, derived from *B anthina* DSM 16086 | This study |
| *B. anthina* S3 | Glyphosate-resistant suppressor, derived from *B anthina* DSM 16086 | This study |
| *B. anthina* S4 | Glyphosate-resistant suppressor, derived from *B anthina* DSM 16086 | This study |
| *B. anthina* S5 | Glyphosate-resistant suppressor, derived from *B anthina* DSM 16086 | This study |
| *B. anthina* S6 | Glyphosate-resistant suppressor, derived from *B anthina* DSM 16086 | This study |
| *B. anthina* S7 | Glyphosate-resistant suppressor, derived from *B anthina* DSM 16086 | This study |
| *B. anthina* S8 | Glyphosate-resistant suppressor, derived from *B anthina* DSM 16086 | This study |
| *B. anthina* S9 | Glyphosate-resistant suppressor, derived from *B anthina* DSM 16086 | This study |
| *B. anthina* S2.1 | Glyphosate-resistant suppressor, derived from S2 | This study |
| *Escherichia coli* XL1-Blue | *recA1 endA1 gyrA96 thi-1 hsdR17 supE44 relA1 lac* [F*’* *proAB lacI*^q^ *Z*∆*M15* Tn*10* (*Tet^r^*)] | Stratagene |
| *Escherichia coli* DH5α | f80d*lacZ* DM15 *recA1 endA1 gyrA96 relA1 thi-1* *hsdR17(r_k_^-^m_k_^-^) supE44 deoR* Δ*(lacZYA-argF) U169* | Laboratory strain collection |
| *Escherichia coli* BL21(DE3) | F^–^ *ompT* *gal* *dcm* *lon* *hsdS_B_*(*r_B_*^–^*m_B_*^–^) λ(DE3 [*lacI* *lacUV*5-*T7p07* *ind*1 *sam*7 *nin*5]) [*malB*^+^]_K-12_(λ^S^) | Stratagene |
| *Escherichia coli* BTH101 | F^-^ *cya-99 araD139 galE15 galK16 rpsL1* (*Str^r^*) *hsdR2*   *mcrA1 mcrB1* | Euromedex; Karimova et al., 1998 |
| *Escherichia coli* W3110 | **-** | Laboratory strain collection |
| **Primers** | **Sequence 5’ -> 3’** | **Purpose** |
| CM1 | AAATCTAGAGATGACTAACGCAGCAAACG | B2H assay; amplification of *ppsA^Ba^,* ligation to pKT25, pKNT25, pUT18 and pUT18C via *Kpn*I/*Xba*I |
| CM2 | TTTGGTACCCGCTTCCGGTTCGCCAG | B2H assay; amplification of *ppsA^Ba^,* ligation to pKT25, pKNT25, pUT18 and pUT18C via *Kpn*I/*Xba*I |
| CM3 | AAATCTAGAGATGCTGCCTACCGTTTTC | B2H assay; amplification of *ppsR^Ba^,* ligation to pKT25, pKNT25, pUT18 and pUT18C via *Kpn*I/*Xba*I |
| CM4 | TTTGGTACCCGGTACGACTGCCGGTC | B2H assay; amplification of *ppsR^Ba^,* ligation to pKT25, pKNT25, pUT18 and pUT18C via *Kpn*I/*Xba*I |
| IS59 | AAATCTAGAGATGTCCAACAATGGCT | B2H assay; amplification of *ppsA^Ec^,* ligation to pKT25, pKNT25, pUT18 and pUT18C via *Kpn*I/*Xba*I |
| IS60 | TTTGGTACCC GTTTCTTCAG TTCAGCCA | B2H assay; amplification of *ppsA^Ec^,* ligation to pKT25, pKNT25, pUT18 and pUT18C via *Kpn*I/*Xba*I |
| IS61 | AAATCTAGAGATGGATAATG CTGTTGATC | B2H assay; amplification of *ppsR^Ec^* and *ppsR^Ec^* A518C*,* ligation to pKT25, pKNT25, pUT18 and pUT18C via *Kpn*I/*Xba*I |
| IS62 | TTTGGTACCCGGTACATTCG GCGACTAA | B2H assay; amplification of *ppsR^Ec^* and *ppsR^Ec^* A518C*,* ligation to pKT25, pKNT25, pUT18 and pUT18C via *Kpn*I/*Xba*I |
| IS47 | AAACTGCAGATGTCCAACAATGGCT | Construction of pBP1224; amplification of *ppsA^Ec^,* ligation to pGP380 via *Pst*I/*Hind*III |
| IS48 | TTTAAGCTTTTATTTCTTCAGTTCAGCC | Construction of pBP1224; amplification of *ppsA^Ec^,* ligation to pGP380 via *Pst*I/*Hind*III |
| IS49 | AAAGAGCTCGATGTCCAACAATGGC | Construction of pBP1225; amplification of *ppsA^Ec^,* ligation to pGP172 via *Sac*I/*Bam*HI |
| IS50 | TTTGGATCCTTATTTCTTCAGTTCAGCC | Construction of pBP1225; amplification of *ppsA^Ec^,* ligation to pGP172 via *Sac*I/*Bam*HI |
| IS51 | AAACTGCAGATGGATAATGCTGTTGATC | Construction of pBP1226 and pBP1227; amplification of *ppsR^Ec^,* ligation to pWH844 via *Pst*I/*Hind*III |
| IS52 | TTTAAGCTTTCTAGTACATTCGGCGA | Construction of pBP1226 and pBP1227; amplification of *ppsR^Ec^,* ligation to pWH844 via *Pst*I/*Hind*III |
| IS63 | AAACTGGCAATGCCGTTTGGTATCCGCGC | Mutagenesis of *ppsR* A518C (Q173P) |
| IS64 | TTTGATACCAAACGGCATTGCCAGATACAG AC | Mutagenesis of *ppsR* A518C (Q173P) |
| IS55 | ACGACGTTCAGATACGACTC | Sequencing primers for *ppsR* |
| IS56 | GAATGGAGTCGCTGAATGTC | Sequencing primers for *ppsR* |
| **Plasmids** | **Construction, purpose** | **Reference** |
| pGP172 | P*_T7_-*mcs *bla*; expression of N-terminally Strep-tagged proteins in *E. coli* BL21(DE) | Merzbacher et al., 2004 |
| pKT25 | P*_lac_-cyaT25-*mcs *aphA3*; protein-protein interaction analysis, B2H assay | Karimova et al., 1998 |
| pKNT25 | P*_lac_-*mcs*-cyaT25* *aphA3*; Protein-protein interaction analysis, B2H assay | Karimova et al., 1998 |
| pUT18C | P*_lac_-cyaT18-*mcs *bla*; protein-protein interaction analysis, B2H assay | Karimova et al., 1998 |
| pUT18 | P*_lac_-*mcs*-cyaT18* *bla*; protein-protein interaction analysis, B2H assay | Karimova et al., 1998 |
| pKT25-zip | P*_lac_-cyaT25-*yeast GCN4 leucine zipper *aphA3*; protein-protein interaction analysis, B2H assay | Karimova et al., 1998 |
| pUT18C-zip | P*_lac_-cyaT18-*yeast GCN4 leucine zipper *bla*; protein-protein interaction analysis, B2H assay | Karimova et al., 1998 |
| pWH844 | P*_T5_-*mcs *bla*; expression of N-terminally His-tagged proteins in *E. coli* DH5α | Schirmer et al., 1997 |
| pGP380 | P*_deqQ_ ermC* and *bla*; expression of genes in *Bacillus subtilis* and *Escherichia coli* | Herzberg et al., 2007 |
| pBP78 | pKT25-*ppsA^Ec^*, expression of T25-PpsA in *E. coli* BTH101 | This study |
| pBP79 | pKNT25-*ppsA^Ec^*, expression of PpsA-T25 in *E. coli* BTH101 | This study |
| pBP80 | pUT18-*ppsA^Ec^*, expression of PpsA-T18 in *E. coli* BTH101 | This study |
| pBP81 | pUT18C-*ppsA^Ec^*, expression of T18-PpsA in *E. coli* BTH101 | This study |
| pBP82 | pKT25-*ppsR^Ec^*, expression of T25-PpsR in *E. coli* BTH101 | This study |
| pBP83 | pKNT25-*ppsR^Ec^*, expression of PpsR-T25 in *E. coli* BTH101 | This study |
| pBP84 | pUT18-*ppsR^Ec^*, expression of PpsR-T18 in *E. coli* BTH101 | This study |
| pBP85 | pUT18C-*ppsR^Ec^*, expression of T18-PpsR in *E. coli* BTH101 | This study |
| pBP1207 | pKT25-*ppsR^Ba^*, expression of T25-PpsR in *E. coli* BTH101 | This study |
| pBP1208 | pKNT25-*ppsR^Ba^*, expression of PpsR-T25 in *E. coli* BTH101 | This study |
| pBP1209 | pUT18-*ppsR^Ba^*, expression of PpsR-T18 in *E. coli* BTH101 | This study |
| pBP1210 | pUT18C-*ppsR^Ba^*, expression of T18-PpsR in *E. coli* BTH101 | This study |
| pBP1211 | pKT25-*ppsA^Ba^*, expression of T25-PpsA in *E. coli* BTH101 | This study |
| pBP1212 | pKNT25-*ppsA^Ba^*, expression of PpsA-T25 in *E. coli* BTH101 | This study |
| pBP1213 | pUT18-*ppsA^Ba^*, expression of PpsA-T18 in *E. coli* BTH101 | This study |
| pBP1214 | pUT18C-*ppsA^Ba^*, expression of T18-PpsA in *E. coli* BTH101 | This study |
| pBP1224 | pGP380-*ppsA^Ec^*, overexpression PpsA from *E. coli* in *E. coli* and *B. subtilis* | This study |
| pBP1225 | pGP172-*ppsA^Ec^*, expression of N-terminally Strep-tagged PpsA from *E. coli* in *E. coli* BL21(DE) | This study |
| pBP1226 | pWH844-*ppsR^Ec^*, expression of N-terminally 6 x His-tagged PpsR from *E. coli* in *E. coli* BL21(DE) | This study |
| pBP1227 | pWH844-*ppsR^Ec^* A518C, expression of N-terminally 6 x His -tagged PpsR Q173P from *E. coli* in *E. coli* BL21(DE) | This study |
| pBP1228 | pKT25-*ppsR^Ec^* A518C, expression of T25-PpsR Q173P in *E. coli* BTH101 | This study |
| pBP1229 | pKNT25-*ppsR^Ec^* A518C, expression of PpsR Q173P-T25 in *E. coli* BTH101 | This study |
| pBP1230 | pUT18-*ppsR^Ec^* A518C, expression of PpsR Q173P-T18 in *E. coli* BTH101 | This study |
| pBP1231 | pUT18C-*ppsR^Ec^* A518C, expression of T18-PpsR Q173P in *E. coli* BTH101 | This study |

**
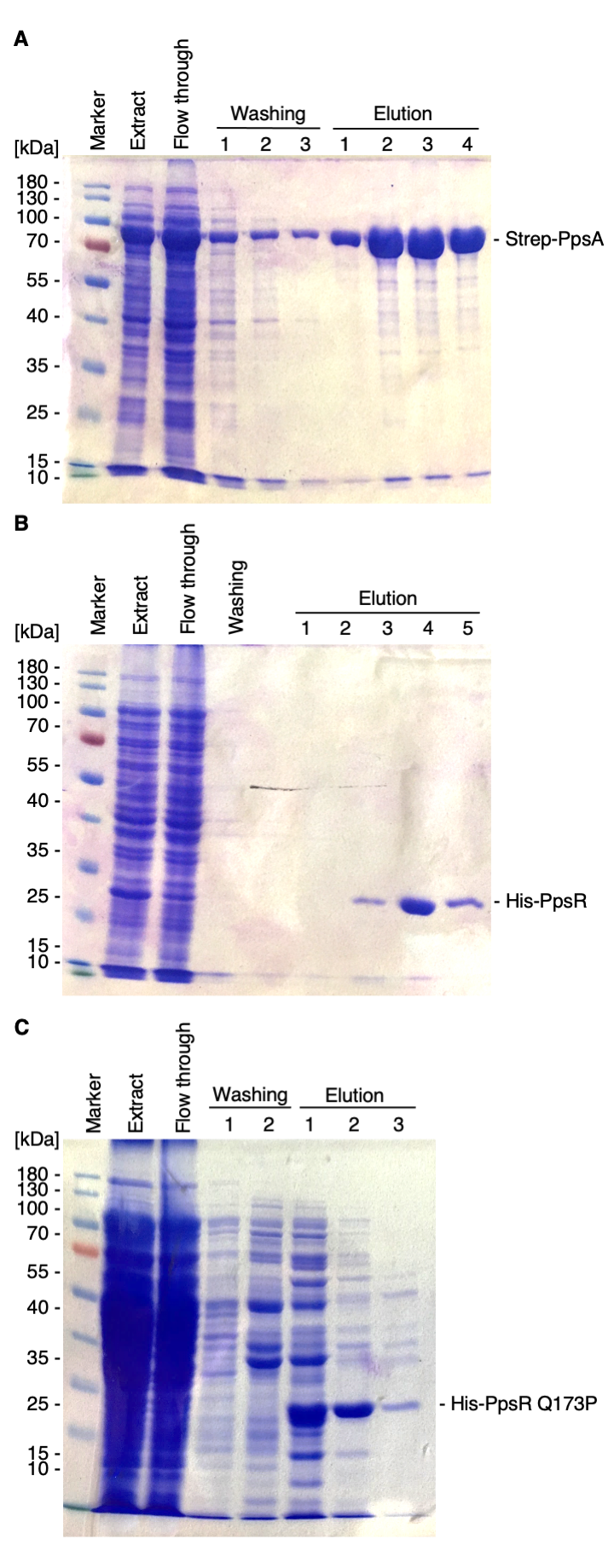
**

**Fig. S1.** **Purification of *E. coli* PpsA, PpsR and PpsR Q173P.**

Analysis of the fractions obtained during purification of Strep-PpsA (A), His-PpsR (B) and His-PpsR Q173P (C).The proteins in 10 µl of the fractions were analysed by 12.5% SDS PAGE and the proteins were visualized by Coomassie staining. The PageRuler Prestained Protein Ladder (Thermofisher, Germany) served as a marker.

**Fig. S2.** **Effect of *ppsA* overexpression on GS resistance in *B. subtilis* and *E. coli.***

For the drop dilution assay, the *B. subtilis* and *E. coli* strains carrying the plasmids pGP380 (- PpsA) and pBP1224 (+ PpsA) were grown over night in LB medium at 37°C. and the cultures were used to inoculate C-Glc medium. After 6 h of growth at 37°C, the cells were washed twice in C salts, the OD_600_ was adjusted to 1 and 5 µl of the diluted cells were spotted onto C agar plates supplemented with either 0.5% (w/v) glucose or 0.6% (w/v) pyruvate. Ammonium served as the nitrogen source. The plates were incubated for 36 h at 37°C.

**References**

Herzberg C, Weidinger LAF, Dörrbecker B, Hübner S, Stülke J, Commichau FM. 2007. SPINE: a method for the rapid detection and analysis of protein-protein interactions *in vivo*. Proteomics 7: 4032-4035.

Karimova G, Pidoux J, Ullmann A, Landant D. 1998. A bacterial two-hybrid system based on a reconstituted signal transduction pathway. Proc Natl Acad Sci USA 95: 5752-5756.

Merzbacher M, Detsch C, Hillen W, Stülke J. 2004. *Mycoplasma pneumoniae* HPr kinase/phosphorylase. Eur J Biochem 271: 367-374.

Richts B, Hertel R, Potot S, Poehlein A, Daniel R, Schyns G, Prágai Z, Commichau FM. 2020. Complete genome sequence of the prototrophic *Bacillus subtilis* subsp. *subtilis* strain SP1. Microbiol Resourc announc 9: e00825-20.

Schirmer F, Ehrt S, Hillen W. 1997. Expression, inducer spectrum, domain structure, and function of MopR, the regulator of phenol degradation in *Acinetobacter calcoaceticus* NCIB8250. J Bacteriol 179: 1329-1336.

Vandamme P, Henry D, Coenye T, Nzula S, Vancanneyt M, LiPuma JJ, Speert DP, Govan JRW, Mahenthiralingam E. 2002. *Burkholderia anthina* sp. nov. and *Burkholderia pyrrocinia,* two additional *Burkholderia cepacian* complex bacteria, may confound results of new molecular diagnostic tools. FEMS Immunol Med Microbiol 33: 143-149.
